# Pharmacological inhibition of Bcl-xL sensitizes osteosarcoma to doxorubicin

**DOI:** 10.18632/oncotarget.5333

**Published:** 2015-09-25

**Authors:** Zuzanna Baranski, Yvonne de Jong, Trayana Ilkova, Elisabeth F.P. Peterse, Anne-Marie Cleton-Jansen, Bob van de Water, Pancras C.W. Hogendoorn, Judith V.M.G. Bovée, Erik H.J. Danen

**Affiliations:** ^1^ Division of Toxicology, Leiden/Academic Center for Drug Research, Leiden University, 2300 RA Leiden, The Netherlands; ^2^ Department of Pathology, Leiden University Medical Center, 2300 RC Leiden, The Netherlands

**Keywords:** osteosarcoma, apoptosis, autophagy, Bcl-xL, doxorubicin

## Abstract

High-grade conventional osteosarcoma is the most common primary bone tumor. Prognosis for osteosarcoma patients is poor and resistance to chemotherapy is common. We performed an siRNA screen targeting members of the Bcl-2 family in human osteosarcoma cell lines to identify critical regulators of osteosarcoma cell survival. Silencing the anti-apoptotic family member Bcl-xL but also the pro-apoptotic member Bak using a SMARTpool of siRNAs as well as 4/4 individual siRNAs caused loss of viability. Loss of Bak impaired cell cycle progression and triggered autophagy. Instead, silencing Bcl-xL induced apoptotic cell death. Bcl-xL was expressed in clinical osteosarcoma samples but mRNA or protein levels did not significantly correlate with therapy response or survival. Nevertheless, pharmacological inhibition of a range of Bcl-2 family members showed that inhibitors targeting Bcl-xL synergistically enhanced the response to the chemotherapeutic agent, doxorubicin. Indeed, in osteosarcoma cells strongly expressing Bcl-xL, the Bcl-xL-selective BH3 mimetic, WEHI-539 potently enhanced apoptosis in the presence of low doses of doxorubicin. Our results identify Bcl-xL as a candidate drug target for sensitization to chemotherapy in patients with osteosarcoma.

## INTRODUCTION

Osteosarcoma is the most common primary malignant bone tumor occurring predominantly in the second decade of life, and a second peak at middle age. It is thought to arise from mesenchymal stem cells that can produce osteoid [1–3]. About 30–40% of the patients with localized osteosarcoma will relapse mainly by presenting with lung metastasis. Approximately 10–20% of the patients present with metastasis at the moment of diagnosis. Since the introduction of chemotherapy patients with local disease have 50–60% long-term survival rate. There has been no significant further improvement over the past three decades [4]. Following disease relapse, prognosis is very poor with 23–33% 5-year overall survival despite repeated metastasectomies when feasible [5].

Apoptosis is a form of programmed cell death, which requires caspase-mediated proteolysis, and is governed by the Bcl-2 family. It is essential for development and tissue homeostasis, and can mediate cell death upon exposure to pathogens, cytotoxic agents, or oncogenic stress [6]. The Bcl-2 family includes BH3-only proteins (Bim, Puma, Bad, Noxa, Bik, Hrk, Bmf and tBid), pro-survival proteins (Bcl-2, Bcl-xL, Bcl-w, Mcl-1, Bfl-1, and Bcl-B) and pro-apoptosis proteins (Bax, Bak and Bok). Bok is primarily localized to ER and Golgi membranes where it was found to be important for a proper ER stress response. Its overexpression induces apoptosis in a manner that is dependent on Bax and Bak [7]. Bak is localized to mitochondria and Bax resides in the cytosol. Once Bak and Bax are activated, they undergo conformational changes and Bax localizes to the mitochondria. In the mitochondria, Bak and Bax form hetero and oligomers which lead to mitochondrial outer membrane permeabilization and cytochrome-c release, which is necessary for caspase activation [8]. Under normal conditions, the pro-survival Bcl-2 members form heterodimers with Bax or Bak inhibiting their activation. However, under cytotoxic stress the activated BH3-only proteins displace these proteins allowing Bax and Bak to cause cytochrome-c release from the mitochondria, caspase cascade activation, and ultimately cell death [8, 9].

Impaired apoptosis is one of the hallmarks of cancer [10]. It allows cancer cells to tolerate oncogenic stress and survive in hostile environments such as hypoxic conditions. Furthermore, defects in apoptosis in cancer cells can hamper the response to chemotherapy [11]. In this study we used RNA interference and pharmacological inhibition to identify members of the Bcl-2 family that control osteosarcoma cell survival and resistance to chemotherapy.

## RESULTS

### Identification of Bcl-xL as a critical pro-survival factor in osteosarcoma cells

**Figure 1:**
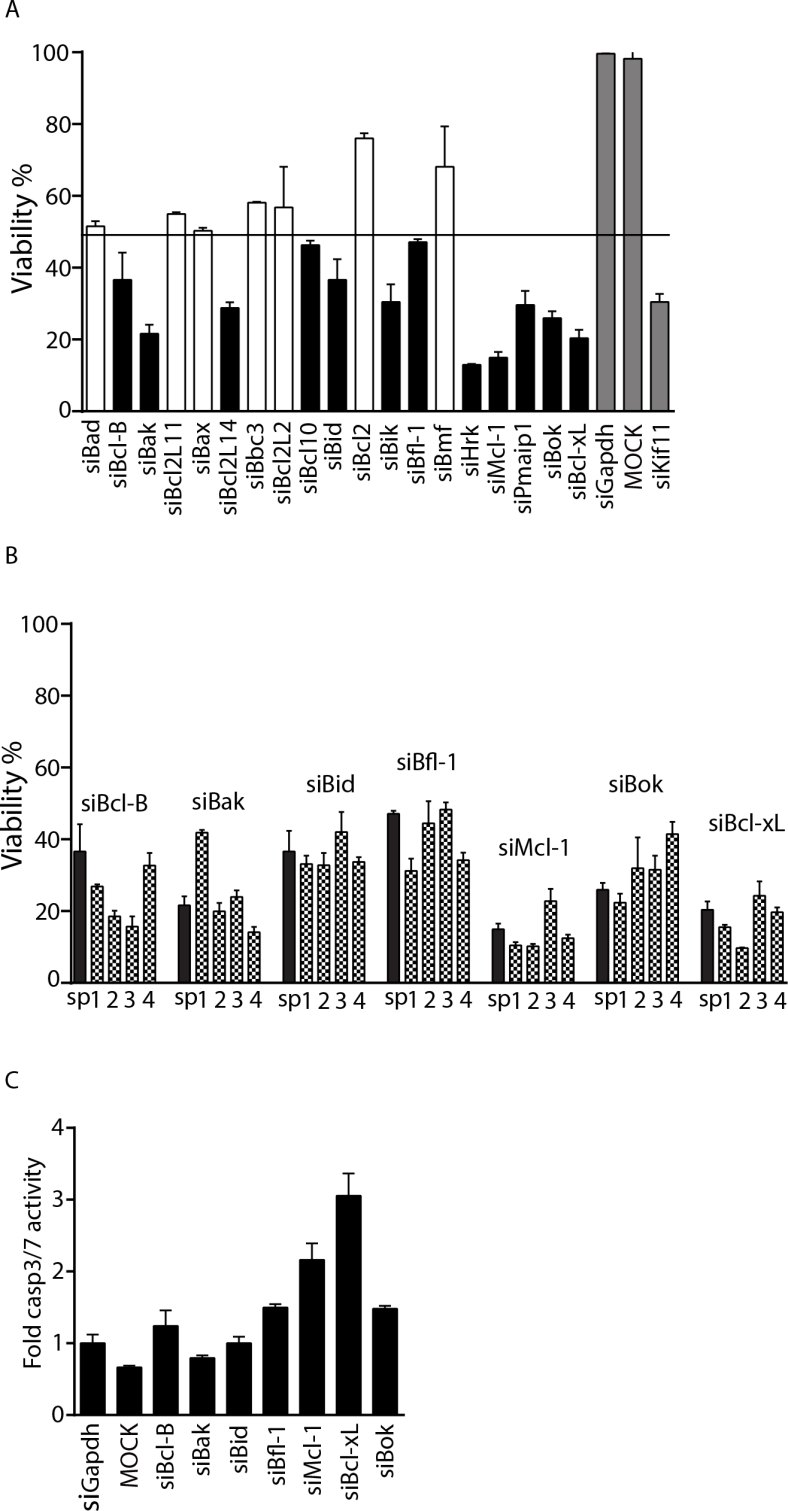
Identification of Bcl-xL as a candidate target for sensitization to doxorubicin **A.** Average U2OS viability in wells transfected with siRNA SMARTpools targeting the indicated genes relative to siGapdh. Mean and standard deviation is shown. Horizontal line marks 2 SD threshold (<48.4% viability), hits are indicated in black, positive and negative controls in grey. **B.** Validated hits where all 4 single siRNAs mimic the SMARTpool and are below the threshold. Black bars represent the SMARTpool and grey patterned bars represent the single siRNAs. **C.** Caspase3/7 activity in U2OS cells transfected with SMARTpool siRNAs targeting the indicated genes. Values were normalized to control siGapdh. One representative experiment of two performed in quadruplicate is shown. Error bars represent standard deviation.

An siRNA screen was performed in U2OS cells to identify Bcl-2 family members required for osteosarcoma cell viability (See Supplementary Figure S1 for screen layout and results). The screen was performed in duplicate with two negative controls, siGapdh and MOCK (only transfection reagent), and siKif11 as positive control (all controls were present in triplicate in each plate). Each value was normalized to siGapdh, which was set as 100% viability. MOCK transfected cells showed the same viability as siGapdh. To select hits causing loss of viability we calculated the standard deviation across each plate, and determined that a gene was a hit if the SMARTpool (comprised of 4 single siRNAs targeting the same gene) and the 4 single siRNAs tested individually, each were two standard deviations separated from siGapdh. Using this criteria 7 genes were selected, which included Bcl-B, Bak, Bid, Bfl-1, Mcl-1, Bok and Bcl-xL (Figure 1A and 1B). To assess whether loss of viability was due to apoptosis, a caspase 3/7 assay was performed. Caspase 3 and 7 are effector caspases that once activated, lead to cell death by cleaving important structural proteins, and causing DNA fragmentation and membrane blebbing [12]. Bcl-xL knockdown caused the highest caspase3/7 activity after 48 hours of transfection indicating that it effectively triggered apoptosis (Figure 1C).

### Silencing Bak leads to autophagy in osteosarcoma cells

**Figure 2:**
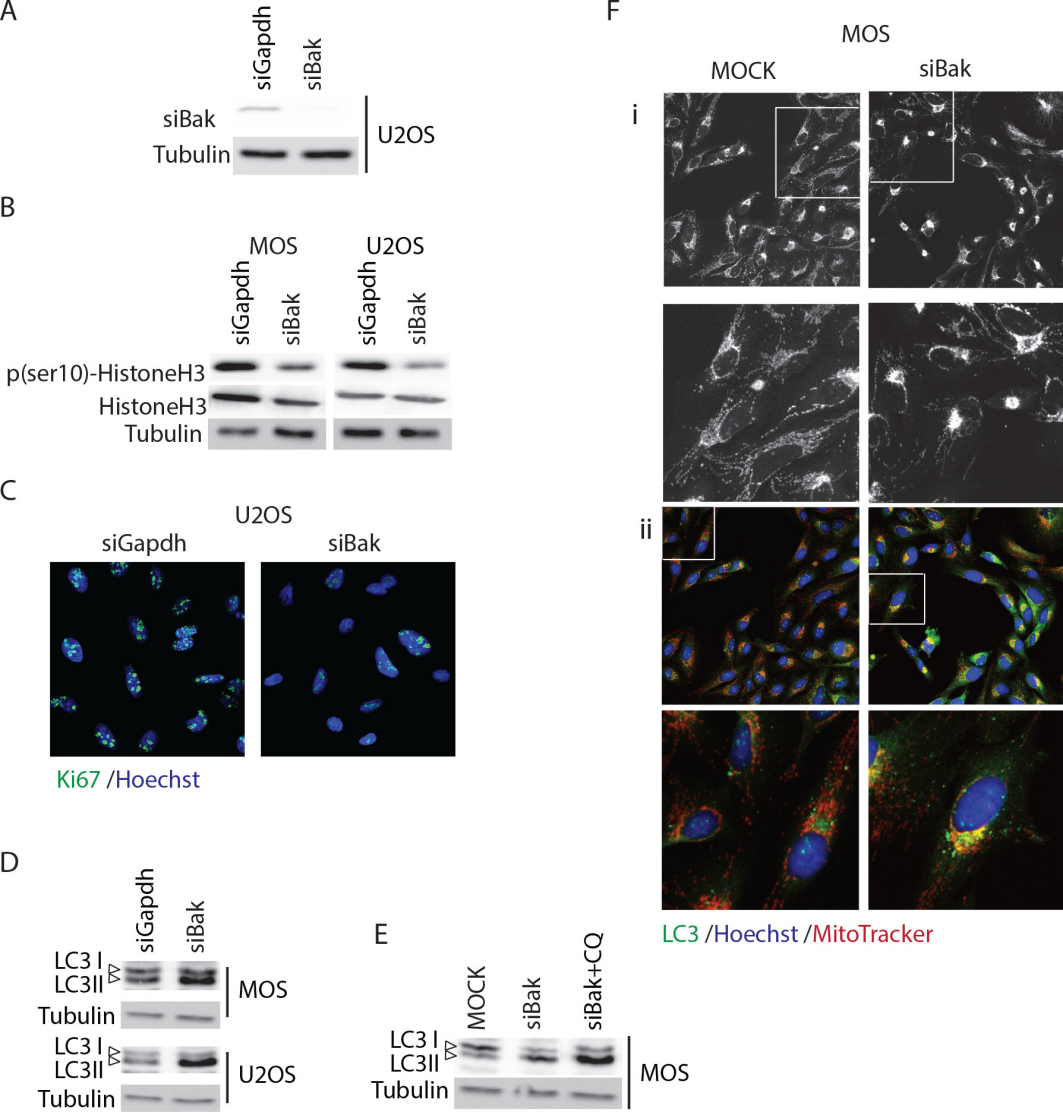
Silencing Bak triggers autophagy in osteosarcoma cells **A.** Western blot analysis of Bak knockdown efficiency in U2OS cells. **B.** Western blot analysis of phospho(ser10)-HistoneH3, total HistoneH3, and tubulin loading control in U2OS and MOS cells transfected with control or Bak siRNA. **C.** Ki67 staining in control (siGapdh) and siBak transfected U2OS cells 48 hours post-transfection (Ki67 antibody, green; Hoechst 33342, blue. **D.** Western blot analysis of LC3 showing LC3-I (upper band) and –II forms in MOS and U2OS cells transiently transfected with siGapdh or siBak SMARTpools. **E.** Western blot analysis of LC3 in control U2OS cells (MOCK) or U2OS transiently transfected with siBak SMARTpool and treated with or without 10 μM chloroquine for 4 hours. **F.** Control MOS cells (MOCK) or MOS cells transiently transfected with siBak for 48 hours, exposed to MitoTracker^®^ (red) for 45 min, fixed and stained with anti-LC3 (green), and Hoechst 33342 (blue). i, MitoTracker only; ii, merge of all three channels.

Surprisingly, the screen also identified pro-apoptotic Bcl-2 family members, including Bak, Bid and Bok (Figure 1A–1B). A caspase activity assay indicated that the loss of cell viability caused by siRNAs targeting these genes was not due to apoptosis (Figure 1C). It has been described that failure to activate apoptosis in Bak/Bax double knockout cells is accompanied by increased autophagy [13, 14]. Autophagy is a recycling process that provides building blocks and energy during cell stress while unlimited autophagy leads to cell death [15]. We first assessed the knockdown efficiency of Bak in U2OS cells and confirmed a ~100% efficacy (Figure 2A). Since there was no caspase 3/7 activation in response to Bak knockdown, we next determined if the observed loss of cells was due to slower proliferation. Phospho(ser10)-HistoneH3, a marker for cells in mitosis and Ki67, a marker for proliferating cells were analyzed in cell lysates and by immunocytochemistry, respectively. Silencing Bak in U2OS or MOS cells caused a reduction in phospho(ser10)-HistoneH3 levels (Figure 2B) and attenuated proliferation was confirmed by reduced Ki67 staining in si-Bak treated cells (Figure 2C).

To assess if Bak depletion triggered autophagy as shown in other systems [11, 12], we determined the conjugation of the LC3 protein to phosphatidylethanolamine. This conjugation represents a critical step in the formation of the autophagosome, a double-membrane organelle that engulfs cellular components during autophagy and subsequently fuses with the lysosome [16]. Silencing Bak in U2OS and in MOS cells led to accumulation of the conjugated form of LC3, termed LC3-II (Figure 2D). Moreover, treatment of U2OS cells in which Bak was silenced with 10 μM chloroquine for 4 hours (chloroquine acidifies the phagosome inhibiting its fusion with the lysosome leading to autophagosome accumulation [17]) led to further accumulation of LC3 II (Figure 2E). In addition, immunocytochemistry showed that mitochondria no longer distributed throughout the cytoplasm but clustered perinuclearly in Bak-depleted cells where they colocalized with LC3-marked autophagosomes (Figure 2F). Together, these findings indicate that depletion of Bak in osteosarcoma cells induces altered mitochondrial distribution, decreased proliferation, and autophagy.

### Bcl-xL is expressed in osteosarcoma lung metastasis

**Figure 3:**
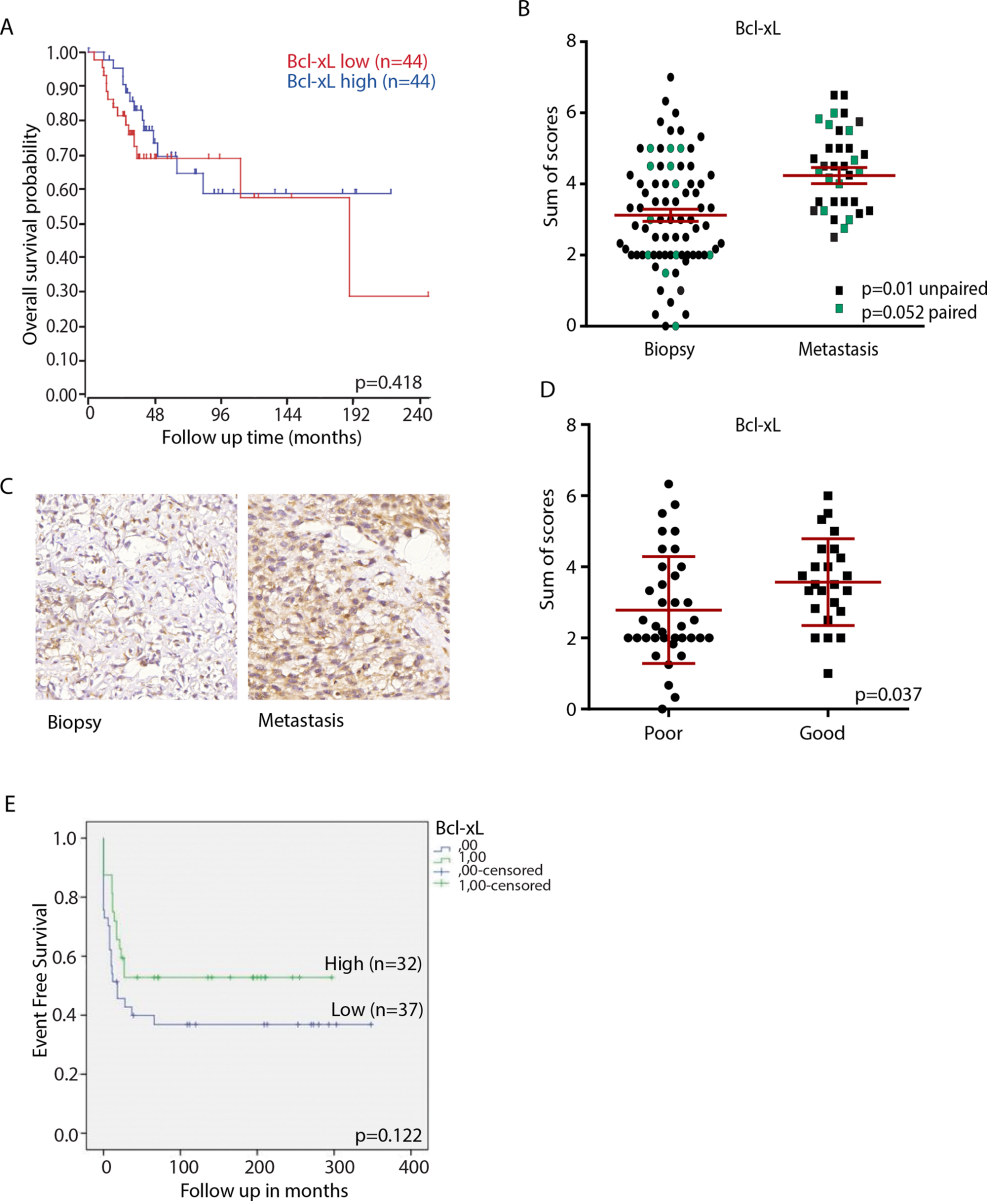
Bcl-xL expression in osteosarcoma clinical samples **A.** Kaplan–Meijer curve showing relation between Bcl-xL mRNA expression level and overall survival in a panel of 88 osteosarcoma patients. The cohort was divided into high and low expression at the median. The curve was made using http://r2.amc.nl. *p* value was determined by Bonferroni testing. **B.** Sum score (% plus intensity of staining) of Bcl-xL expression in all tumors included in the tissue microarray; average is shown in red. Biopsies and metastases from the same patient are indicated in green (*n* = 12); *p*-value determined by paired *t*-test. Unpaired biopsies (*n* = 59) and metastases (*n* = 7) are indicated in black; *p*-value determined by unpaired *t*-test. **C.** Representative images of Bcl-xL expression in primary osteosarcoma biopsy and metastasis. Images made with 40x Lens. **D.** Bcl-xL expression in biopsies of poor and good responders to chemotherapy. *P*-value determined by unpaired two-tailed *t*-test. **E.** Event free survival related to Bcl-xL expression in tissue microarray. Tumors were divided according to low (mean sum score ≤3; blue line (*n* = 40)) or high Bcl-xL expression (mean sum score >3; green line (*n* = 29)). *p*-value determined by Log-rank test.

As Bcl-xL silencing caused severe loss of viability and led to the strongest induction of caspase 3/7 activity, we decided to further study its role in osteosarcoma. Others have reported that high Bcl-xL mRNA expression in osteosarcoma patients is correlated with lower overall survival rate [18]. We analyzed Bcl-xL mRNA expression using a previously published microarray data set of a cohort of 88 osteosarcoma patients but did not observe significant association with overall survival (Figure 3A). We next analyzed the expression of Bcl-xL by immunohistochemistry in 60 human primary osteosarcomas and 23 osteosarcoma lung metastases. Expression of Bcl-xL was detected in the majority of osteosarcoma samples and expression was higher in metastases compared to primary tumors (Figure 3B, 3C). However, contrary to our expectations, high Bcl-xL protein expression levels (SUM score of intensity and % positive cells) in primary biopsies correlated with good response to therapy in this study (>90% necrosis post-chemotherapy) (Figure 3D). Furthermore, event-free survival rates did not significantly differ between patients with high and low Bcl-xL expression in diagnostic biopsies (Figure 3E). These results indicate that Bcl-xL is expressed in advanced osteosarcomas, but its expression is not correlated with poor therapy response or survival.

### Pharmacological inhibition of Bcl-xL sensitizes osteosarcoma cells to chemotherapy

**Figure 4:**
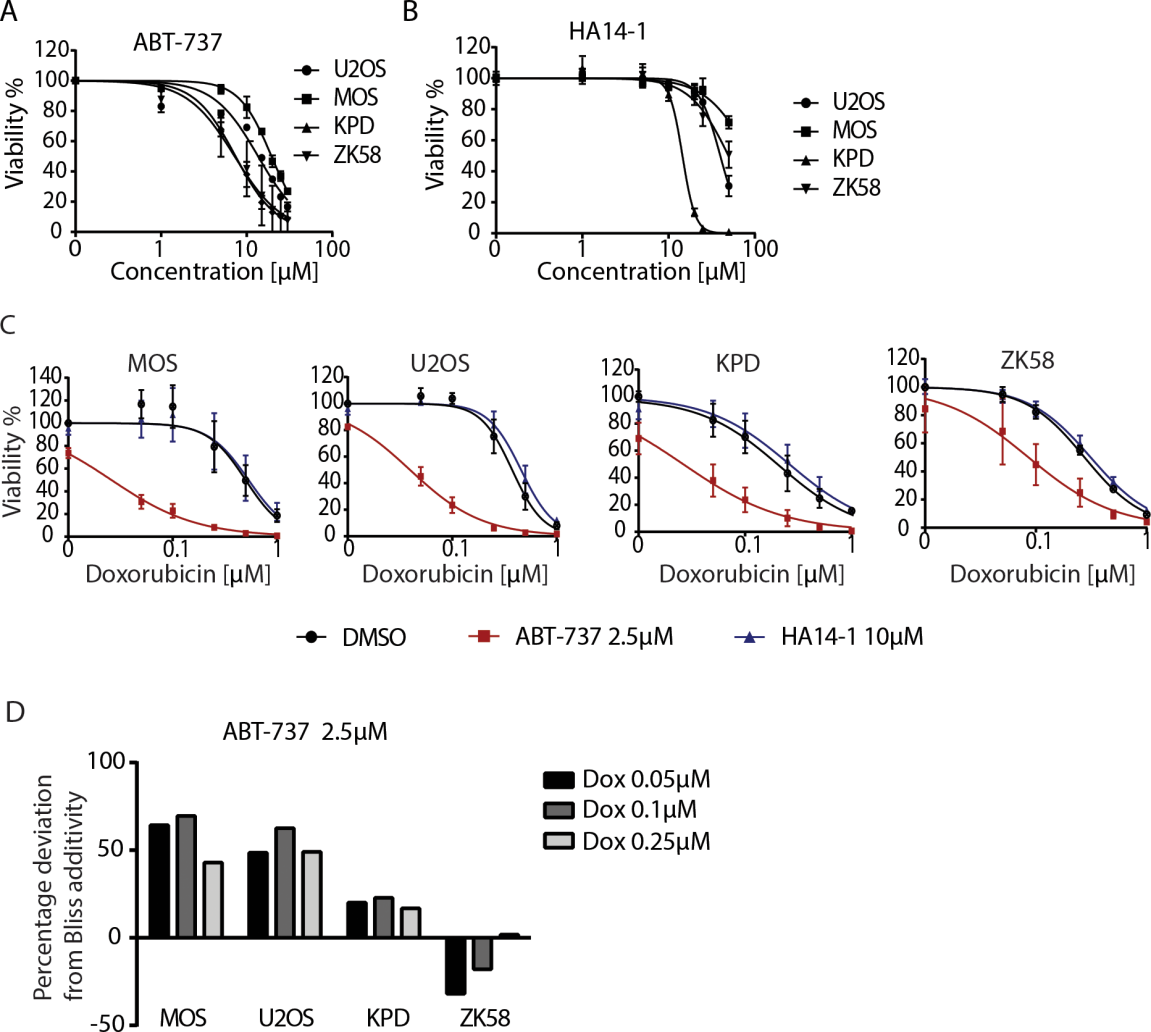
Pharmacological inhibition of Bcl-xL sensitizes osteosarcoma cells to chemotherapy **A, B.** Dose response curve for ABT-737 (A) and HA14-1 (B) in the indicated human osteosarcoma cell lines. Error bars represent the standard deviation of two experiments performed in triplicate. Cells were exposed for 72 hours. **C.** Dose response curves for doxorubicin in the indicated osteosarcoma cell lines in absence (black circle) or presence of 2.5 μM ABT-737 (red square) or 10 μM HA14-1 (blue triangle). Cells were exposed for 72 hours. Error bars represent mean ± SEM of three experiments. **D.** Bar graph presenting the percentage of deviation from Bliss additivity based on data shown in C. One representative of three independent experiments is shown.

Although we could not confirm earlier observations correlating Bcl-xL expression to patient survival, our siRNA screen indicated that Bcl-xL inhibition might enhance tumor killing in cases where it is expressed. Therefore, we assessed whether Bcl-xL represents a relevant target for osteosarcoma alone or in the context of conventional chemotherapy. First, four human osteosarcoma cell lines were treated with ABT-737, a BH3 mimetic that inhibits Bcl-w, Bcl-xL and Bcl-2; of which only Bcl-xL was identified in the siRNA screen (Figure 1A). In addition, HA14-1, a selective Bcl-2 inhibitor was tested. All the cell lines showed loss of cell viability in the 10 μM range for ABT-737 and at higher concentrations for HA14-1 (Figure 4A and 4B). Subsequently, we asked if these inhibitors, when used at lower concentrations, could sensitize osteosarcoma cells to chemotherapy. The four osteosarcoma cell lines were co-treated with a suboptimal concentration of ABT-737 and a dose range of doxorubicin for 72 hours. At a concentration of 2.5 μM of ABT-737, viability of the cells was close to 100%. MOS, U2OS and to a lesser extent KPD and ZK58 showed increased sensitivity to treatment with 50–500 nM doxorubicin under these conditions (Figure 4C). By contrast, treatment with up to 10 μM of the Bcl-2 selective inhibitor, HA14-1 did not affect sensitivity to doxorubicin. Indeed, calculation of the deviation from additivity as predicted by Bliss independence model [19] indicated synergy between ABT-737 and doxorubicin (Figure 4D).

**Figure 5:**
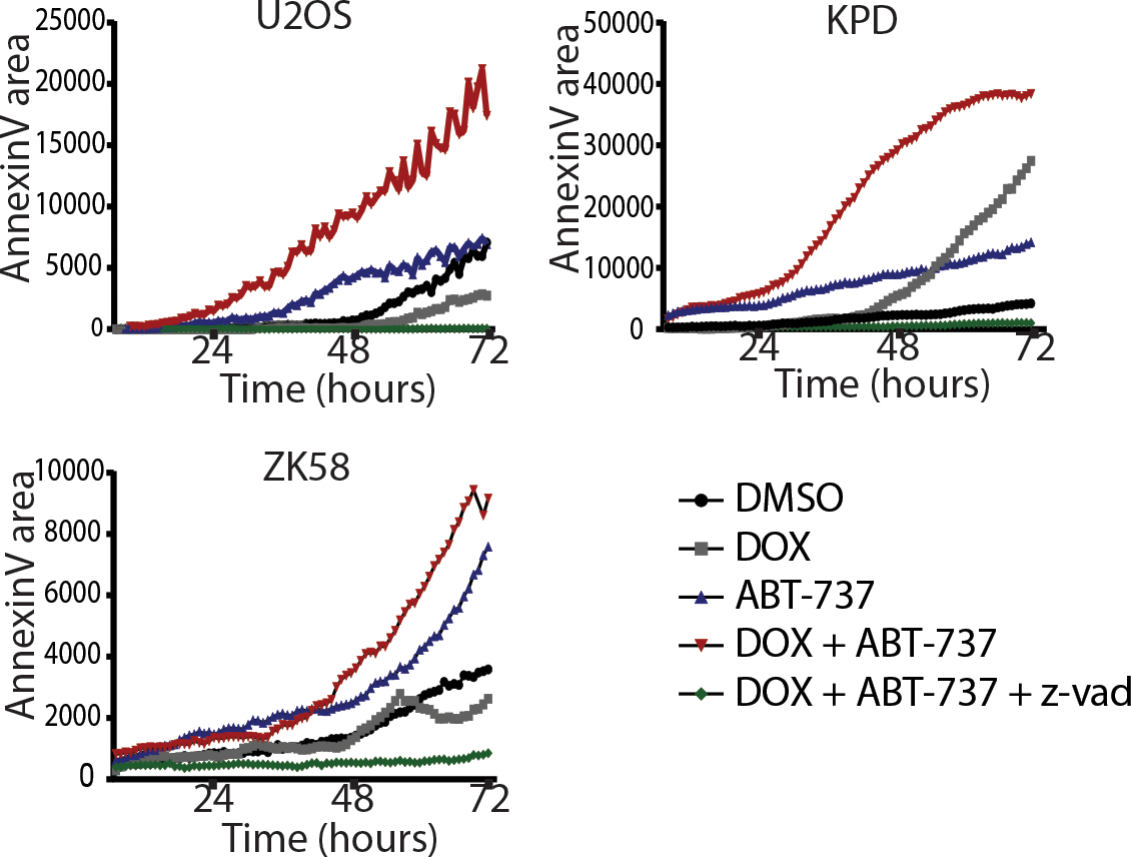
Inhibition of Bcl-xL in combination with doxorubicin leads to enhanced apoptosis Live imaging of Annexin V accumulation in MOS, U2OS and ZK58 cells. Cells were treated with DMSO, 0.1 μM Doxorubicin, 2.5 μM ABT-737, or the combination with or without zvad-fmk as indicated. Graphs represent one of three independent experiments. Annexin V was quantified with ImagePro Analyzer 7.0.

We next determined if synergy was due to enhanced apoptosis in the presence of the combination of ABT-737 and doxorubicin. For this purpose, real time imaging was used to detect labeled Annexin V binding to phosphatidylserine, a phospholipid that translocates to the outer lipid layer of the membrane when cells enter apoptosis [20]. In agreement with the viability assays (Figure 4C), ZK58 cells showed significant Annexin V labeling already in response to 2.5 μM ABT-737 alone (Figure 5). However, in U2OS and KPD cells exposed to 2.5 μM ABT-737 or 0.1 μM doxorubicin alone, Annexin V labeling was absent or appeared at late time points after exposure whereas the combination of 2.5 μM ABT-737 and 0.1 μM doxorubicin caused rapid, strong Annexin V labeling. Moreover, this enhanced response to the combination of ABT-737 and chemotherapy was abolished in the presence of the pan-caspase inhibitor z-VAD-fmk (Figure 5). These results indicate that ABT-737, but not the Bcl-2 selective inhibitor HA 14–1 can sensitize human osteosarcoma cells to chemotherapy leading to enhanced apoptosis.

### Inhibition of Bcl-xL with WEHI-539 sensitizes osteosarcoma to doxorubicin

**Figure 6:**
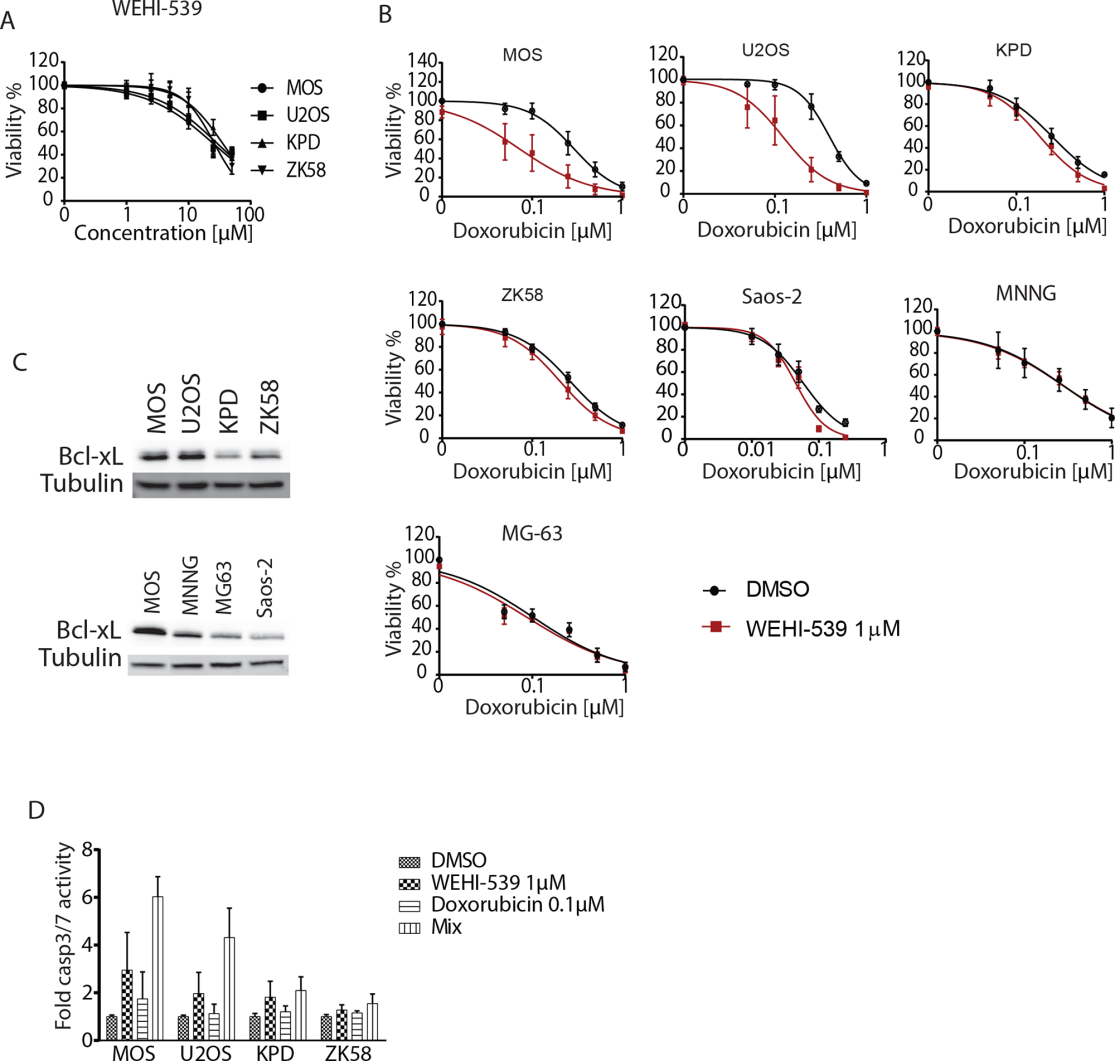
Inhibition of Bcl-xL with WEHI-539 sensitizes osteosarcoma to doxorubicin **A.** WEHI-539 dose response curves for the indicated human osteosarcoma cell lines. Cells were exposed for 72 hours. Error bars represent standard deviation of two experiments performed in triplicate. **B.** Doxorubicin dose response curves for the indicated osteosarcoma cell lines in absence (black circles) or presence of 1 μM WEHI-539 (red squares). Cells were exposed for 72 hours. Mean ± SEM of three experiments is shown. **C.** Western blot analysis of Bcl-xL expression in the indicated osteosarcoma cell lines. **D.** Caspase 3/7 activity in the indicated osteosarcoma cell lines exposed to DMSO, 0.1 μM doxorubicin, 1 μM WEHI539 or the combination (mix). Mean ± SEM of three experiments is shown.

To further pinpoint the sensitization to chemotherapy to inhibition of Bcl-xL, we made use of a recently developed Bcl-xL-selective BH3 mimetic, WEHI-539 [21]. Exposing U2OS and MOS cells to this compound caused loss of viability at concentrations between 1–10 μM and a similar response was seen in KPD and ZK58 cells at concentrations >10 μM (Figure 6A). Moreover, a suboptimal dose of 1 μM WEHI-539 effectively enhanced the response to low doses of doxorubicin in U2OS and MOS cells but showed no effect in KPD, ZK58, MNNG, MG-63 and Saos-2. (Figure 6B). This difference could be attributed to differences in Bcl-xL expression: Bcl-xL protein levels were high in U2OS and MOS as compared to the other cell lines (Figure 6C). Lastly, we investigated whether sensitization to doxorubicin in the presence of WEHI-539 was due to enhanced apoptosis. Indeed, MOS and U2OS cells exposed to 1 μM WEHI-539 showed 2–3-fold higher induction of caspase 3/7 activity in response to 0.1 μM doxorubicin, which by itself had little or no effect (Figure 6D). In agreement with the absence of synergy observed in KPD and ZK58 (Figure 6B), WEHI-539 failed to increase caspase activation in response to doxorubicin in these cells (Figure 6D). Altogether, these findings demonstrate that osteosarcoma cells expressing Bcl-xL can be sensitized to chemotherapy through pharmacological Bcl-xL inhibition.

## DISCUSSION

Patients with metastatic or recurrent osteosarcoma present low probability of survival mainly due to resistance to standard chemotherapy [22, 23]. Bcl-2 family proteins play a crucial role in regulating cell survival/cell death pathways, and aberrations in their expression or function mediates tumor development and progression [24]. Our siRNA screen identifies anti-apoptosis genes such as Bcl-xL, Mcl-1 and Bfl-1, but interestingly, it also identified pro-apoptosis genes such as Bak, Bok and Bid. Autophagy was reported to be enhanced in Bak/Bax double knockout cells in response to death stimuli such as radiation and cytotoxic drugs [25, 26]. Others have associated cell cycle arrest with Bak/Bax double knockout conditions [27, 28]. We identify both aspects in Bak-silenced osteosarcoma cells: the cells proliferate slower and autophagy is activated. We also notice a recruitment of mitochondria to the perinuclear area where they colocalize with autophagosomes, which may point to mitoautophagy. Alternatively, this may reflect the recent demonstration that mitochondria can in fact contribute to the formation of the autophagosome membrane [29].

Bcl-xL expression has been associated with poor prognosis for patients with colorectal cancer [30] and hepatocellular carcinoma [31]. In colorectal cancer, high expression of Bcl-xL correlates with lymph node metastasis and poorer survival [32]. In melanoma the expression of Bcl-xL is correlated with tumor thickness and disease free survival [33], and in follicular lymphoma it is correlated with overall survival [34]. Bcl-xL expression has also been associated with resistance to cytotoxic agents in ovarian cancer [35]. Furthermore, the correlation between Bcl-xL expression and chemoresistance has been demonstrated in the NCI panel of 60 cell lines to be independent of p53 status [36].

In osteosarcoma, patients with high Bcl-xL mRNA levels have been reported to have a lower probability of 5-year overall survival [18]. We were not able to reproduce such a correlation in our dataset. Moreover, although in our study Bcl-xL protein was expressed in osteosarcoma biopsies and levels were higher in metastases, no correlation with poor response to chemotherapy was observed. The reason for this difference between our own observation and that of others [18] is currently unknown. Nevertheless, in line with the common role of anti-apoptotic Bcl-2 family members in cancer resistance to cytotoxic therapy [37, 38] we find that Bcl-xL inhibition can sensitize osteosarcoma cells to low dose chemotherapy.

ABT-737, a small molecule inhibitor of Bcl-xL, Bcl-2 and Bcl-w, was discovered in 2005 and found to enhance the cytotoxic effect of chemotherapy and radiation [39]. Studies in myeloma cells [40], myeloid leukemias [41], neck-squamous cell carcinoma [42], gastrointestinal stromal tumor cells [43], and chondrosarcoma [38] indicated that ABT-737 treatment sensitizes to chemotherapy and other cytotoxic agents [44]. We show that a similar strategy may be relevant in the context of human osteosarcoma. However, a major limitation for translation to the clinic is the fact that ABT-737 is not orally bioavailable, which limits flexibility of dosing regimens. ABT-263 is an analogue of ABT-737 with oral bioavailability, which also potently inhibits Bcl-xL, Bcl-2 and Bcl-w [45]. It has been demonstrated to have little activity as a single agent in a phase II clinical trial [46] but to enhance the effect of other cytotoxic agents [47–49]. The Bcl-xL-selective BH3 mimetic, WEHI-539 used in our current study has shown *in vivo* toxicity that hinders further clinical studies. Recently, a related Bcl-xL-selective inhibitor, A-1155463, has been synthesized lacking the toxic moiety [50]. Based on our current study, it will be of particular interest to assess how such a pharmacological inhibitor affects the sensitivity of chemoresistant osteosarcomas. High Bcl-xL protein expression as detected by IHC may serve as a biomarker for treatment with Bcl-xL inhibitors alone or in the presence of chemotherapy. Such a strategy, would potentially allow reduced dosage of doxorubicin, thereby decreasing toxicity. Follow up preclinical studies, for instance using xenograft models, will have to determine efficacy and toxicity associated with different concentrations of Bcl-xL inhibitors, doxorubicin, and combinations. Our findings suggest that such studies are warranted to open the possibility for further clinical studies in patients with osteosarcoma.

## MATERIALS AND METHODS

### Reagents and antibodies

Doxorubicin was obtained from the Pharmacy at the Leiden University Academic Hospital, ABT-737, UMI-77 and HA14-1 were from SelleckChem (Huissen, Netherlands). WEHI-539 was from ApexBio (Texas, U.S.A.). The pan-caspase inhibitor z-VAD-fmk was obtained from Bachem (Weil am Rhein, Germany). The Bcl-xL antibody (clone 54H6) used for immunohistochemistry, the Bcl-xL antibody (2762s) used for Western blot and Bak antibody were from Cell Signaling (Bioké, Leiden, The Netherlands). LC3 antibody was from Novus Biologics (Cambridge, England) and Ki67 was from Abcam (Cambridge, England). Chloroquine was bought from Sigma Aldrich (Zwijndrecht, The Netherlands). Hoechst 33342 was purchased from Fischer Scientific (Bleiswijk, The Netherlands).

### Immunohistochemistry on tissue microarrays

Two tissue microarrays used in this study were previously constructed with one was previously published [3]. The second tissue microarray consisted of 73 FFPE biopsies, resections and metastases mainly from high grade conventional osteosarcomas. All specimens in this study were handled according to the ethical guidelines described in ‘Code for Proper Secondary Use of Human Tissue in The Netherlands’ of the Dutch Federation of Medical Scientific Societies. The slide was deparaffinized, rehydrated and blocked of endogenouse peroxidase. Subsequently, antigen retrieval was performed with citrate pH 6.0. Incubation with antibody was overnight at 4°C at a 1:1000 dilution. As a second step we used Immunologic Poly-HRP-GAM/R/R IgG (DVPO110HRP) and Dako liquid DAB+ Substrate Chromogen System (K3468), followed by counterstaining with hematoxylin. Testis tissue was used as control. Slides were scored independently by two observers (JVMGB and YJ). Staining intensity (0 = absent, 1 = weak, 2 = moderate, 3 = strong) and extent of the staining (0 = 0%, 1 = 1–24%, 2 = 25–49%, 3 = 50–74% and 4 = 75–100%) were assessed. The two values were added to obtain sum scores. Cores where the tissue was lost were excluded from the analysis. To assess response to chemotherapy, patients were divided among good and poor responders. The histological response was assessed by determining the amount of necrotic tissue in the resection specimen obtained after chemotherapy. Response was considered good when a patient presented more than 90% necrotic tissue in the tumor, and bad responders were those with less than 90% necrosis [51].

### Cell culture

Human osteosarcoma cell lines MOS, U2OS, 143B, ZK58, KPD, MNNG, MG-63 and Saos-2 were previously described [52, 53]. Cells were grown in RPMI1640 medium supplemented with 10% fetal bovine serum and 25 U/mL penicillin and 25 μg/mL of penicillin-streptomycin. All cells were cultured in a humidified incubator at 37°C with 5% CO_2_.

### siRNA screen

Transient knockdown of individual genes in U2OS cells was achieved using siRNAs from Dharmacon, GE (Landsmeer, Netherlands). The end concentration of siRNA was 20 nM and it was delivered to the cells with INTERFERin siRNA transfection reagent by reverse transfection according to the manufacturer's procedures (Polyplus transfection, Leusden, Netherlands). The transfection was performed in u-clear 96-well plates from Corning. Nineteen Bcl-2 family members were targeted with a SMARTpool comprised of 4 different siRNAs and with each single siRNA individually. After 24 hours of transfection the medium was refreshed and the cells were further incubated for 72 hours. Alamar Blue was acquired from Thermo Fisher Scientific (Bleiswijk, The Netherlands) and used to assess viability as specified by the manufacturer. Fluorescence was measured with a FluoStar Optima plate reader.

### Microarray data analysis

Gene expression profiles were obtained from a previously published microarray data set [54]. Using the Bioconductor lumi package, data was transformed with the variance stabilizing transformation algorithm and normalized with the robust spline normalization algorithm. Probe_ID identifiers from the Illumina Annotation for Illumina human-6 v2.0 expression beadchip were used as reporters (Bcl-xL reporter = ILMN_1654118). Kaplan Meier curves were created from the entry “Mixed Osteosarcoma-Kuijer-127-vst-ilmnhwg6v2” in the web application R2 (http://r2.amc.nl).

### Immunoblotting

Cells were lysed with SDS protein buffer (125 mM Tris/HCl pH 6.8, 20% glycerol, 4% SDS and 0.2% bromophenol blue). Proteins were resolved by SDS-PAGE and transferred to polyvinylidine difluoride membrane. Membranes were blocked in 5% BSA-TBST (TRIS-0.05% Tween20), followed by overnight incubation with primary antibodies and 45 minutes incubation with HRP-conjugated secondary antibodies. Chemoluminescence was detected with the bioimager LAS400 (GE Healthcare).

### Immunostaining

MOS and U2OS cells were transfected with siRNA as previously described. The cells were fixed 48 hours after transfection with ice cold methanol for 15 minutes, and were subsequently rinsed 3 times for 5 minutes with PBS. Afterwards, the cells were incubated with blocking solution [10% normal goat serum, 0.3%Triton100 in PBS] for 1 hour, rinsed 3 times for 5 minutes with PBS, and then incubated 1 hour with second antibody(1:300). Nuclei stainig with Hoechst 33342 was performed as a final step together with the rinsing steps. The antibodies were diluted in antibody staining solution [1%BSA, 0.3%Triton100 in PBS]. The cells probed mitochondria, were exposed to 75 nM of MitoTracker^®^ Red CMX Ros (from Cell Signaling) for 45 minutes previous to fixation. All images were taken with confocal microscope Eclipse Ti-E from Nikon.

### Cell viability, and real time apoptosis assays

For cell viability assays (excluding the siRNA screen), cells were processed using the ATPlite 1Step kit (Perkin Elmer) according to the manufacturer's instructions, followed by luminescence measurement in a Fluostar Optima plate reader. Apoptosis was assessed by Annexin V staining and Caspase Glo3/7 (Promega). For real time Annexin V binding assays, cells were co-exposed to drugs and Annexin V in a *u*-clear 96-well plate, and imaged every hour, for 66 hours. Images were obtained using a BD Pathway 855, then converted to videos, and Annexin V staining was quantified by an in-house macro for Image-Pro Analyzer 7.0 as previously described [20]. For caspase 3/7 activity measurements, cells were exposed to the drug for 24 hours after which the reagent was added 1:1. Luminescence was measured in a Fluostar Optima plate reader.

### Synergy assessment

To assess synergy, we used the Bliss independence model, which defines to what extent the effect of a drug at a certain concentration is independent of the presence of the other drug at a certain concentration [55]. This model predicts the combined response *C* for two single compounds with effects *A* and *B*: *C* = *A*+*B* – A*B [56].

### Statistical analysis

Dose response curve fitting and all statistical analyses were performed with GraphPad Prism 5.0 (GraphPad Software, La Jolla, CA). The unpaired and paired two-tailed *t*-test used to compare groups in Figure 3B and 3D was performed with IBM SPSS statistics 20. Event free survival was computed from the date of diagnosis until first recurrence, either local or metastatic. Tumors were divided into two groups, having low (mean sum score ≤ 3) or high Bcl-xL expression (mean sum score > 3). Event free survival in both groups was compared using the Kaplan-Meier method and the Log-rank test with IBM SPSS statistics 20.

## SUPPLEMENTARY FIGURE


