# Supplementary material for: Pharmacological inhibition of Bcl-xL sensitizes osteosarcoma to doxorubicin
Source: Oncotarget. 2015 Sep 25;6(34):36113–25. doi: 10.18632/oncotarget.5333 (PMC4742165; doi:10.18632/oncotarget.5333)
Supplement: Supplementary file 1 [file oncotarget-06-36113-s001.pdf]

SUPPLEMENTARY FIGURE

siRNA screen layout and results

exp 1 PLATE1_1

A		1078	1056	743	660	587	2047	862	543	404	364	723	mean siGAPDH	2053 μ_n
B		480	849	443	519	312	2048	1122	665	1302	1561	640	mean siKif11	629 μ_p
C		1044	414	457	797	645	2064	614	302	1531	514	322	s.d siGAPDH	9,539392 σ_n
D		1189	1384	1135	935	497	2035	1331	1404	972	2030	385	s.d siKif11	41,01219 σ_p
E		969	449	1197	636	320	2088	836	714	723	782	672		
F		1582	828	947	1957	831	658	696	1340	1178	945	1146	**Z'-factor**	**0,893501**
G		957	690	1002	961	732	600	1562	1437	1653	1761	1415		
H														

PLATE1_2

A		1459	1719	1045	913	1006	2875	904	788	504	396	873	mean siGAPDH	2888 μ_n
B		576	1224	528	656	378	2876	1597	987	1879	2377	852	mean siKif11	823 μ_p
C		1436	626	674	1000	1021	2913	797	408	2122	825	336	s.d siGAPDH	21,65641 σ_n
D		1684	2121	1707	1387	838	2634	1410	1905	1221	3233	477	s.d siKif11	17,67767 σ_p
E		1310	618	1291	1019	373	2736	940	909	877	1329	1001		
F		2166	1237	1190	3157	1164	835	778	1815	1607	1533	1831	**Z'-factor**	**0,94287**
G		1378	833	1160	1436	946	810	1737	2067	2391	2826	2645		
H														

exp 2 PLATE2_1

A		403	1695	700	663	694	3145						mean siGAPDH	3158 μ_n
B		438	311	307	646	369	3171						mean siKif11	653 μ_p
C		848	913	1522	1189	1133	642						s.d siGAPDH	18,38478 σ_n
D		777	652	816	907	1235	661						s.d siKif11	10,01665 σ_p
E		592	506	301	857	593	657							
F													**Z'-factor**	**0,965982**
G														
H														

PLATE2_2

A		396	1942	842	655	692	3074						mean siGAPDH	3019 μ_n
B		485	336	323	761	397	2964						mean siKif11	651 μ_p
C		978	1038	1841	1136	1307	663						s.d siGAPDH	77,78175 σ_n
D		824	730	1148	1035	1324	635						s.d siKif11	14,57166 σ_p
E		665	455	297	645	624	656							
F													**Z'-factor**	**0,882982**
G														
H														

PLATE 1 layout

	1	2	3	4	5	6	7	8	9	10	11	12
A		BAD_sp	BAD_1	BAD_2	BAD_3	BAD_4	siGAPDH	BCL2L10_sp	BCL2L10_1	BCL2L10_2	BCL2L10_3	BCL2L10_4
B		BAK1_sp	BAK1_1	BAK1_2	BAK1_3	BAK1_4	siGAPDH	BCL2L11_sp	BCL2L11_1	BCL2L11_2	BCL2L11_3	BCL2L11_4
C		BAX_sp	BAX_1	BAX_2	BAX_3	BAX_4	siGAPDH	BCL2L14_sp	BCL2L14_1	BCL2L14_2	BCL2L14_3	BCL2L14_4
D		BBC3_sp	BBC3_1	BBC3_2	BBC3_3	BBC3_4	MOCK	BCL2L2_sp	BCL2L2_1	BCL2L2_2	BCL2L2_3	BCL2L2_4
E		BCL10_sp	BCL10_1	BCL10_2	BCL10_3	BCL10_4	MOCK	BID_sp	BID_1	BID_2	BID_3	BID_4
F		BCL2_sp	BCL2_1	BCL2_2	BCL2_3	BCL2_4	Kif11	BIK_sp	BIK_1	BIK_2	BIK_3	BIK_4
G		BCL2A1_sp	BCL2A1_1	BCL2A1_2	BCL2A1_3	BCL2A1_4	Kif11	BMF_sp	BMF_1	BMF_2	BMF_3	BMF_4
H												

PLATE 2 layout

	1	2	3	4	5	6	7	8	9	10	11	12
A		HRK_sp	HRK_1	HRK_2	HRK_3	HRK_4	siGAPDH					
B		MCL1_sp	MCL1_1	MCL1_2	MCL1_3	MCL1_4	siGAPDH					
C		PMAIP1_sp	PMAIP1_1	PMAIP1_2	PMAIP1_3	PMAIP1_4	Kif11					
D		BOK_sp	BOK_1	BOK_2	BOK_3	BOK_4	Kif11					
E		BCL2L1_sp	BCL2L1_1	BCL2L1_2	BCL2L1_3	BCL2L1_4	Kif11					
F												
G												
H												

Supplementary Figure S1: Characterization of the siRNA screen. Raw values and plate layouts for siRNA screen using SMARTpool (sp) and single siRNAs (_1, _2, _3, _4) are shown. Mean and SD for positive (siKif11) and negative controls (siGapdh) are shown on the right and these values were used to calculate the indicated Z' factors according to: $Z' = 1 - 3*(\sigma_p + \sigma_n)/ |\mu_p - \mu_n|$.
